# Attitudes and perceptions of affected women towards endocrine endometriosis therapy: an international survey based on free-word association networks

**DOI:** 10.1093/humrep/dead221

**Published:** 2023-10-25

**Authors:** N Thurnherr, L Burla, J M Metzler, B File, P Imesch

**Affiliations:** Department of Gynecology, University Hospital Zurich, Zurich, Switzerland; Department of Gynecology, University Hospital Zurich, Zurich, Switzerland; Department of Gynecology, University Hospital Zurich, Zurich, Switzerland; Theoretical Neuroscience and Complex Systems Research Group, Department of Computational Sciences, Wigner Research Centre for Physics, Budapest, Hungary; Department of Gynecology, University Hospital Zurich, Zurich, Switzerland

**Keywords:** endometriosis, endometriosis drug therapy, social media, free-word association, endometriosis survey

## Abstract

**STUDY QUESTION:**

What are the attitudes and perceptions towards endocrine endometriosis therapy?

**SUMMARY ANSWER:**

Among the study population, endocrine endometriosis therapies are associated with negative mental images and emotions and there seems to be a pre-therapeutic information deficit on the part of physicians.

**WHAT IS KNOWN ALREADY:**

Endocrine therapies, as the current standard of conservative endometriosis treatment, have good efficacy and improve symptoms and quality of life in most patients. Nevertheless, clinical practice repeatedly shows rejection on the part of patients, which may result in reduced compliance and discontinuation of therapy.

**STUDY DESIGN, SIZE, DURATION:**

Cross-sectional study among endometriosis patients using a multilingual questionnaire distributed via the most popular social media channels between November 2020 and February 2021. A total of 3348 women participated in the study.

**PARTICIPANTS/MATERIALS, SETTING, METHODS:**

Based on a pilot phase, an international, multilingual online survey was conducted among women affected by endometriosis. The questionnaire included free-word associations and questions about personal medical history, source of information, and demographic data. Mental representations were detected based on modules of the co-occurrence network of associations.

**MAIN RESULTS AND THE ROLE OF CHANCE:**

Six modules with different dominant emotional labels emerged from the confluence of associations to endocrine endometriosis therapy mentioned by participants. Five modules reflected negative mental associations, with the most frequently mentioned words being ‘side effects’, ‘pain’, ‘ineffective’, ‘depression’, and ‘uncertainty’. Of the 12 most frequently selected emotions, only ‘optimistic’ was positive. Side effects affecting mental health are the most important reason for deciding against endocrine therapy in our survey population. Twenty-seven percent of respondents reported knowing little about endocrine therapies for endometriosis. Social media are the most frequently used sources of information and were rated as the most useful.

**LIMITATIONS, REASONS FOR CAUTION:**

By translating the questionnaire, questions might have been understood differently depending on the language. By using social media channels for distribution, digitally literate patients were targeted. The survey population might not be representative as patients who are critical/unhappy with therapy are more likely to seek advice from peer groups.

**WIDER IMPLICATIONS OF THE FINDINGS:**

The findings of this study replicate the findings of a recent survey in three European countries. Given the prevalence of endometriosis and the few emerging pharmaceutical alternatives, these data point to a growing need for further research and development of non-hormonal drugs for treating endometriosis. Most endometriosis patients are young and digitally literate, and much information is obtained from alternative sources, such as social media. Careful education before starting therapy should be taken seriously, and patients’ concerns should be addressed individually by health care providers. This could help reduce misunderstanding and misinformation and improve treatment adherence and satisfaction.

**STUDY FUNDING/COMPETING INTEREST(S):**

There is no funding or conflict of interest to declare.

**TRIAL REGISTRATION NUMBER:**

The trial is not registered at any trial registry.

## Introduction

Endometriosis is a chronic inflammatory, oestrogen-dependent disease affecting women of reproductive age. It is defined as endometrial glandular cells and stroma occurring outside the cavum uteri. With an estimated prevalence of at least 10%, endometriosis is one of the most common benign gynaecological disorders. Its exact pathophysiology and aetiogenesis remain poorly understood. The heterogeneous symptoms include dysmenorrhoea, non-cyclical pelvic pain, infertility, dyspareunia, and dyschezia, which often lead to a considerable reduction in the quality of life of the affected patients (Shafrir *et al.*, 2018; Chapron *et al.*, 2019; Zondervan *et al.*, 2020).

Furthermore, endometriosis is associated with high costs for the social and healthcare system, comparable to other chronic diseases (O’Hara *et al.*, 2021; Simoens *et al.*, 2012). Laparoscopy, considered the gold standard for diagnosis, is currently used with restraint. In case of clinical suspicion, medical endocrine therapy is initiated (Chapron *et al.*, 2019; Becker *et al.*, 2022).

As there is currently no pharmaceutical curative treatment, endometriosis therapy aims to relieve pain and ameliorate symptoms (Chapron *et al.*, 2019). Among the nonsurgical options, endocrine drugs represent the therapy of choice, as non-hormonal drug treatment is limited to analgesics and anti-inflammatory substances, such as non-steroidal anti-inflammatory drugs (NSAIDs) (Streuli *et al.*, 2013; Rafique and Decherney, 2017; Chapron *et al.*, 2019). First-line therapy consists of NSAIDs coupled with combined oral contraceptives or progestins. If the therapeutic effect is unsatisfying or insufficient, GnRH analogues, aromatase inhibitors, and GnRH antagonists can be considered (Vercellini *et al.*, 2018; Taylor *et al.*, 2021; Becker *et al.*, 2022).

Research and development of alternative treatments has yielded little success so far (Groothuis and Guo, 2018; Guo and Groothuis, 2018). Most drugs tested in recent or ongoing trials still target endocrine pathways; the rest consists predominantly of repurposed therapeutics, which are unpromising so far (Guo and Groothuis, 2018). Unfortunately, no new therapeutic approach seems to be in the pipeline for the coming decade (Groothuis and Guo, 2018). Researchers have called for a paradigm shift in drug research and development in endometriosis (Guo and Groothuis, 2018). The urgent need for innovation is underlined by a recent survey in three European countries (Austria, Germany, Switzerland), showing that endocrine endometriosis therapeutics are unpopular among most patients and that non-endocrine drugs would be preferred. Apart from side effects, the mere fact of endocrine activity appears to be bothersome (Burla *et al.*, 2021).

At the same time, there are new relevant sources of information and opportunities for exchange with people who are also affected. Most endometriosis patients are in their reproductive phase and digitally literate. There is an abundance of endometriosis-related social media groups and influencers on diverse platforms (Metzler *et al.*, 2022).

Using an open-ended questionnaire technique with free-word associations, we can aim to map the relevant mental representations and discover new dimensions of the perception of endocrine therapy. This method has recently been successfully applied to other chronic diseases (Gero *et al.*, 2020). In this study, we address what accounts for the mistrust of endocrine therapeutic options and what practitioners could do to address it.

## Materials and methods

### Ethical approval

The local ethics committee approved this study prior to its initiation (ID Req-2020-00470). The need for written informed consent was waived. Nevertheless, at the beginning of the questionnaire, there was detailed information about the research process and the aim of a scientific publication, which all the women who participated agreed to.

### Population

This survey targeted individuals who reported receiving a professional diagnosis of endometriosis (either from their gynaecologist, family physician, or surgeon). Individuals whose diagnosis was based solely on their own opinion were excluded. Nevertheless, detailed information about the research process and the aim of the scientific publication was provided at the beginning of the questionnaire, and consent was a prerequisite for participation.

To identify endometriosis-related social media communities on Facebook, Instagram (both Menlo Park, CA, USA), and Twitter (San Francisco, CA, USA), search terms such as ‘endometriosis’ or ‘endo’ were applied. Using the previously established social media profile/hashtag ‘endosurvey_usz’, the weblink to the survey was distributed. Between November 2020 and February 2021, participation reminders were posted repeatedly on all three social media platforms. Additionally, the link was posted in numerous endometriosis-related Facebook groups, and endometriosis Instagram influencers were personally asked to share.

### Questionnaire

Based on a previous survey (Burla *et al.*, 2021), an online questionnaire was initially created as a pilot in English (Qualtrics (Provo, UT, USA)). This included 28 multiple-choice questions with a comment option for participants to suggest improvements. A total of 102 individuals completed the pilot survey. After adaptation, the final questionnaire with 29 multiple-choice questions was developed. The questionnaire was translated into six languages (English, German, French, Italian, Spanish, and Portuguese) by native speakers to ensure accurate and meaningful transcription.

#### Structured surveys

One part of the survey consisted of questions about personal medical history, including past or present endocrine therapy, source of information and demographics (see Supplementary Data File S1 for the full questionnaire). Endocrine drugs were divided into the following groups: combined contraceptives, progestins/gestagens, dienogest, GnRH analogues, GnRH antagonists, aromatase inhibitors, danazol, and long-acting gestagens.

### Free-word association

To explore the focus of this work on patients’ attitudes, emotions, and perceptions towards endocrine treatment of endometriosis, free-word associations were used as the second main part of this questionnaire. The free-word association method was established to objectify the opinions and attitudes of participants towards a specific topic. Participants were asked to name five words that came to their minds while thinking of endocrine treatment for endometriosis. Next, each word had to be linked to two emotions out of a list of 22 (attentive (P), hostile (N), irritable (N), alert (P), ashamed (N), excited (P), guilty (N), enthusiastic (P), distressed (N), determined (P), upset (N), scared (N), afraid (N), interested (P), strong (P), nervous (N), sad (N), frustrated (N), angry (N), disappointed (N), optimistic (P), annoyed (N)). (P = positive; N = negative), in which eight emotions were positive, and 14 emotions were negative. The emotions were based on the Positive and Negative Affect Schedule (PANAS), and refined in the pilot study with the frequently recommended emotions of the respondents (Watson *et al.*, 1988). Using emotions to enhance the analysis of free-word associations has proved beneficial in recent studies (File *et al.*, 2019; Gero *et al.*, 2020).

### Data preprocessing

Free-word associations to ‘endocrine therapy’ were first spellchecked (transformed to lowercase, removed accents, and manually corrected) and lemmatized and translated to English by native speakers in each language. Associations were merged if their English translation was identical or close synonyms.

### Analysis of the association networks

In this analysis, major mental representations of endocrine therapy among women suffering from endometriosis were extracted from the free associations. We could define networks by defining each unique association as a node and co-occurrence relations between the associations as edges. Statistical co-occurrence between every pair of associations was defined as the log likelihood ratio between the maximum likelihood of the observed co-occurrence and the likelihood of assuming statistical independence. Associations were attractive (get a positively weighted edge) if they were mentioned more frequently together than chance and repulsive (get a negatively weighted edge) if they were mentioned less frequently together than chance. Higher deviation from chance indicated higher negative or positive weight. Densely connected subnetworks were identified as modules. These modules reflect different mental representations of endocrine therapy. Modules were defined by consensus clustering from the maximal modularity partition of the network, applying the extended formula for considering negative edges in the network (Gómez *et al.*, 2009; Lancichinetti and Fortunato, 2012). The associations’ emotional labels described the association modules’ emotional features. The relative percentual distribution of the emotions in each module was presented by eliminating the percentual distribution of the emotions in the overall sample. To characterize the resulting modules with the items of the structured questionnaire, each respondent was given a weighting for each module (ranging from 0 to 5) equal to the number of his/her associations linked to each module. Thus, modules with a higher average weight in the population were considered more important representations of endocrine therapy. The details and methodological considerations of the association network analysis were described in our previous paper (File *et al.*, 2019).

### Statistical analysis

Statistical analysis was performed using R (R Core Team, 2021, Vienna, Austria). First, descriptive statistics were used to characterize the survey population. Second, we used Spearman’s rank correlation test to analyse the relationship between demographic/clinical data and the modules extracted from the association networks. To correct for multiple comparisons, *P*-values below 0.01 were considered statistically significant. Spearman’s rank correlation coefficient (ρ) was used to express the strength of the association between two variables.

The work was carried out following the guidelines of the Checklist for Reporting Results of Internet E-Surveys (CHERRIES) (Eysenbach, 2004).

## Results

### Respondents

Of 5259 who started surveys, 3373 individuals (64.14%) completed the questionnaire. The most frequently chosen language was English (52.83% (n = 1782)), followed by German (38.13% (1286)), French (4.74% (160)), and Italian (3.56% (120)). As only a few participants chose Spanish and Portuguese (0.47% (16) and 0.27% (9)), those were excluded from the analysis. Including these small groups would not have significantly affected our study’s statistical power but would have prevented us from examining hypotheses specific to them. A total of 3348 respondents remained.

### Demographic characteristics

The demographic characteristics of the participants are shown in Table 1. Most respondents lived in Europe (58.99% (n = 1975)) and a city or large city (71.38% (2390)). The ethnicity was predominantly Caucasian (55.68% (1864)), and in terms of religious affiliation, most respondents were Christian (47.34% (1585)) or nonreligious (42.14% (1411)). Regarding the highest level of education, 58.65% (1964) had a university degree (Bachelor/Master/PhD/other doctorate or higher). Concerning relationship status, most participants were married/in a cohabiting relationship (50.96% (1706)) or a relationship (27.36% (916)).

**Table 1.** Demographic data (n of respondents).

		%
Age (years) (3343)	<20	2.0
	20–24	12.7
	25–29	22.9
	30–34	26.7
	35–39	18.6
	40–44	11.2
	>44	6.1
Continent of residence (3348)	Africa	3.5
	Asia	3.9
	Europe	59.0
	North America	29.5
	Oceania	4.0
	South America	0.2
Place of residence (3348)	City	47.1
	Large city	24.3
	Smaller municipality, countryside, village	28.6
Ethnicity (3348)	African	1.4
	Caribbean	0.4
	Caucasian	55.7
	East Asian	1.2
	Latino/Hispanic	3.2
	Middle Eastern	1.4
	Mixed	7.4
	Other	26.1
	South Asian	3.2
Religious background (3348)	Buddhist	0.9
	Christian	47.3
	Hindu	1.3
	Jewish	1.0
	Muslim	2.4
	No religion	42.1
	Other religion	4.9
Highest level of education (3348)	Bachelor’s degree	35.4
	High school	19.2
	Less than a high school	1.1
	Master’s degree	19.5
	Other	8.2
	PhD, other doctorate, or higher	3.7
	Trade school	12.8
Relationship status (3348)	Divorced	1.7
	In relationship	27.4
	Married or in a cohabiting relationship	51.0
	Other	0.6
	Separated	0.9
	Single	18.4
	Widowed	0.1

### Personal medical history and source of information

The highest percentage of respondents were diagnosed with endometriosis by histology (laparoscopy and biopsy) (77.36% (2590)). In 20.94% (701) of cases, endometriosis was suspected or diagnosed by a gynaecologist without histological confirmation and by a family doctor in 1.7% (57) of cases. Participants had suffered from symptoms of endometriosis for an average of 9.53 years (standard deviation 6.6 years, range 0–53) before diagnosis and consulted an average of 5.06 clinics/doctors (standard deviation 4.85, range 0–60) before obtaining the diagnosis of endometriosis.

**Figure 1.**
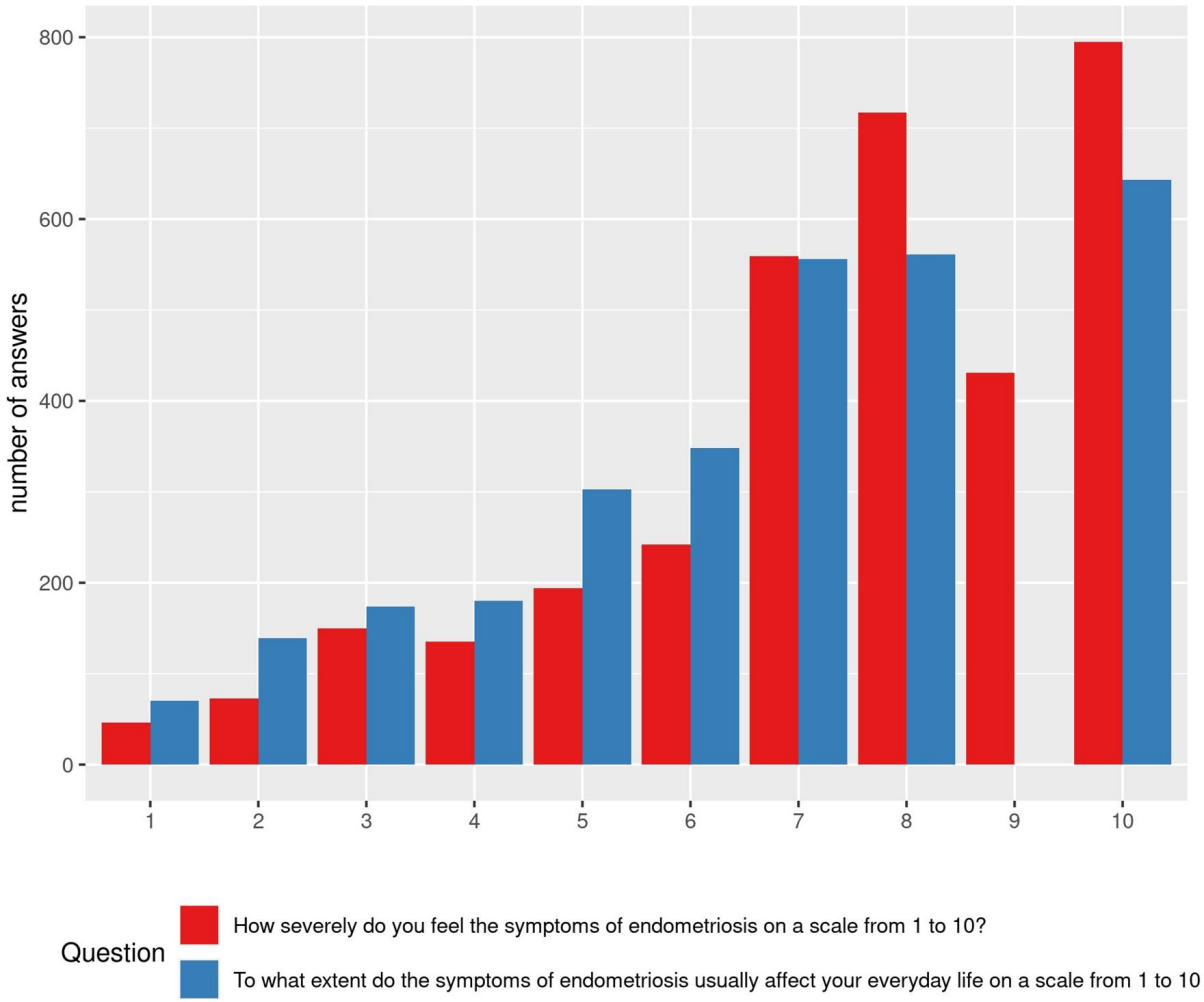
**Severity of endometriosis symptoms (red) and their impact on everyday life (blue).** Horizontal axis shows severity of symptoms/impact (on a scale from 1 to 10) and vertical axis shows relative frequency.

Figure 1 shows the distribution of severity of endometriosis symptoms and their influence on everyday life on a scale from 1 to 10. The largest number of respondents stated to feel severe symptoms of endometriosis (≥7/10) (74.89% (2507)) and their everyday life to be largely affected by those symptoms (≥7/10) (59.17% (1762)).

Most (64.1% (2146)) of the respondents have never been pregnant, and 76.22% (2552) have never given birth. Consequently, 35.9% (1202) have been pregnant in the past (including miscarriages and abortions), and 23.78% (796) have given birth at least once (vaginal birth or Caesarean section).

**Figure 2.**
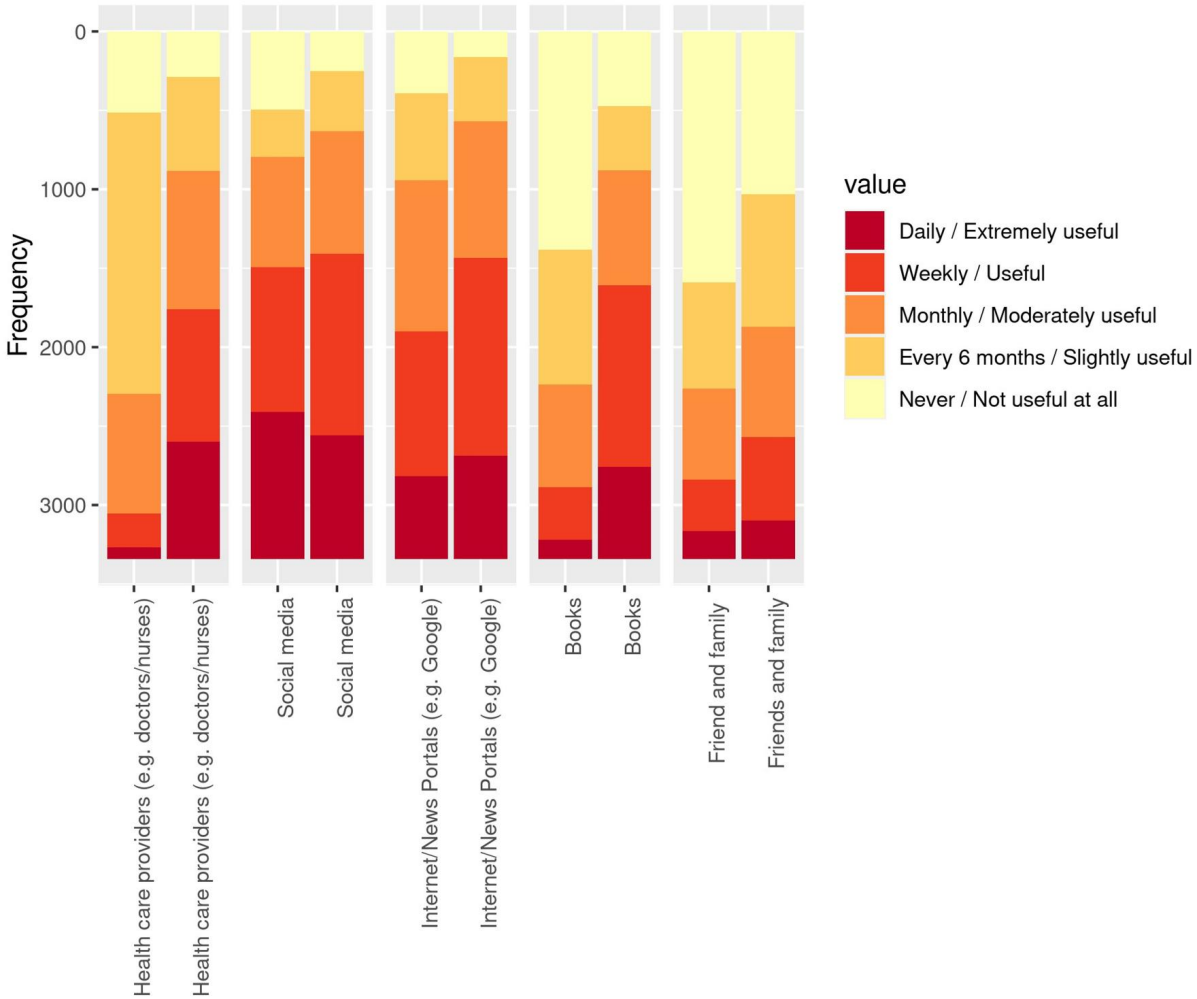
**Sources of information on endometriosis presented based on their popularity (left column in each pair) and usefulness (right column in each pair).** Colour coding shows popularity and usefulness scales. It is important to note that the sources are not ranked but are displayed in the original order as presented in the questionnaire.

Different sources of information ranked by their popularity and usefulness are shown in Fig. 2. Social media were the most frequently used sources among respondents and were also rated the most useful source of information.

### Endocrine therapy

Among respondents, 35.7% (1195) considered themselves well or very well-informed about endocrine therapy. And 37.22% (1246) stated to be informed to a moderate and 21.06% (705) to a small extent. Only 6.03% (202) of participants considered themselves uninformed about endocrine endometriosis therapy. Most (85.9%, n = 2876) respondents had experience with endocrine therapy for endometriosis, and of those, 54.1% (1556) were doing endocrine therapy at the time. Only 14.1% (472) of all participants reported having never done endocrine therapy for endometriosis.

The most used hormonally active drugs were combined contraceptives (73.67% (2118)), followed by dienogest (40.56% (1166), progestins/gestagens (35.37% (1017)), long-acting gestagens (31.37% (902)), GnRH analogues (15.06% (433)), and GnRH antagonists (8.21% (236)). The least popular drugs were Aromatase inhibitors (2.37% (68)) and danazol (1.74% (50)).

Participants who were not doing endocrine therapy or have never done endocrine therapy were mostly not interested in doing endocrine therapy in the future (49.09% (648)). And 18.18% (240) were slightly, and 15.45% (204) were moderately interested in future endocrine therapy. Only 17.28% (228) were either interested or extremely interested in taking endocrine medications in the future.

**Figure 3.**
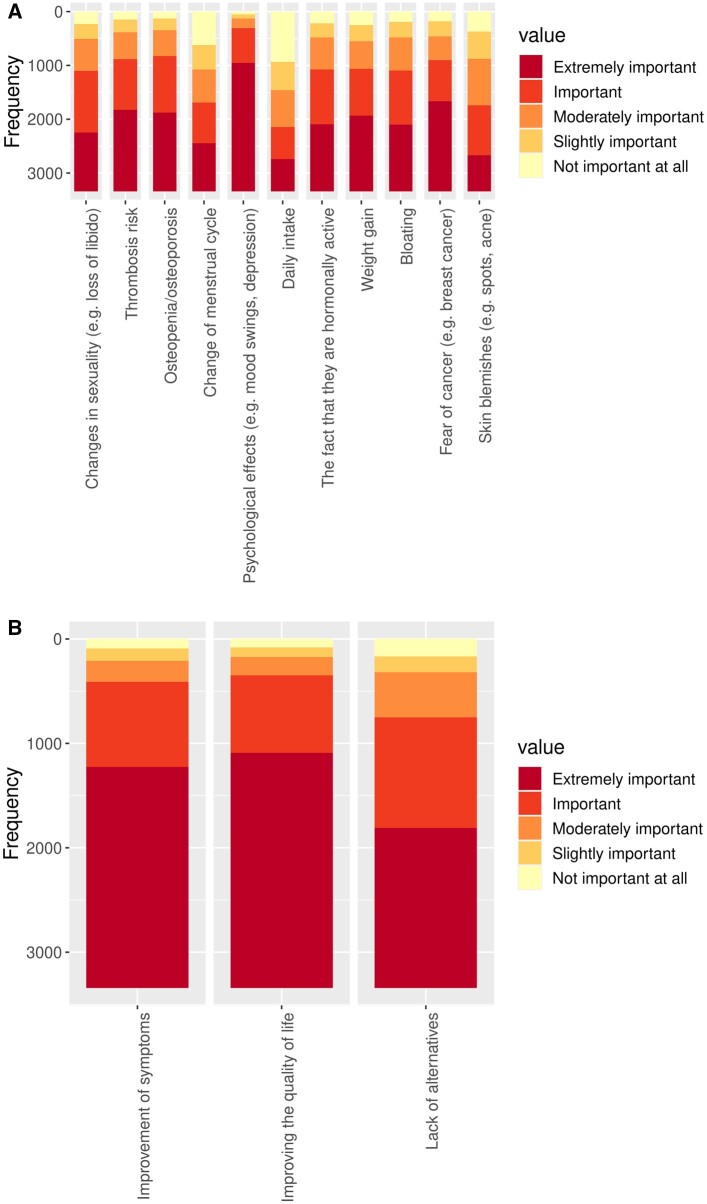
**Distribution of perceived importance for reasons regarding endocrine endometriosis therapy decisions.** (**A**) Reasons for deciding against endocrine endometriosis therapy. (**B**) Reasons for deciding in favour of endocrine endometriosis therapy. Colour coding shows distribution of perceived importance for each reason.

The important reasons for deciding against or in favour of endocrine therapy are shown in Fig. 3. Psychological effects (e.g. mood swings, depression) were considered the most important reason against endocrine therapy, and improvement of quality of life and symptoms seemed to be the most important reasons in favour of endocrine therapy.

### Free-word associations

The total number of lower cased and unaccented unique associations in English, German, French, and Italian were 4865. After the lemmatization, merging, and translation process, the number of associations was 288. Idiosyncratic expressions (associations with 17 occurrences) were excluded, resulting in 165 unique associations in the analysis. The threshold was set based on the function of the cumulative number of excluded associations (Supplementary Fig. S1). Excluding these rarely mentioned associations, only 6% of the produced associations were excluded from the analysis.

**Figure 4.**
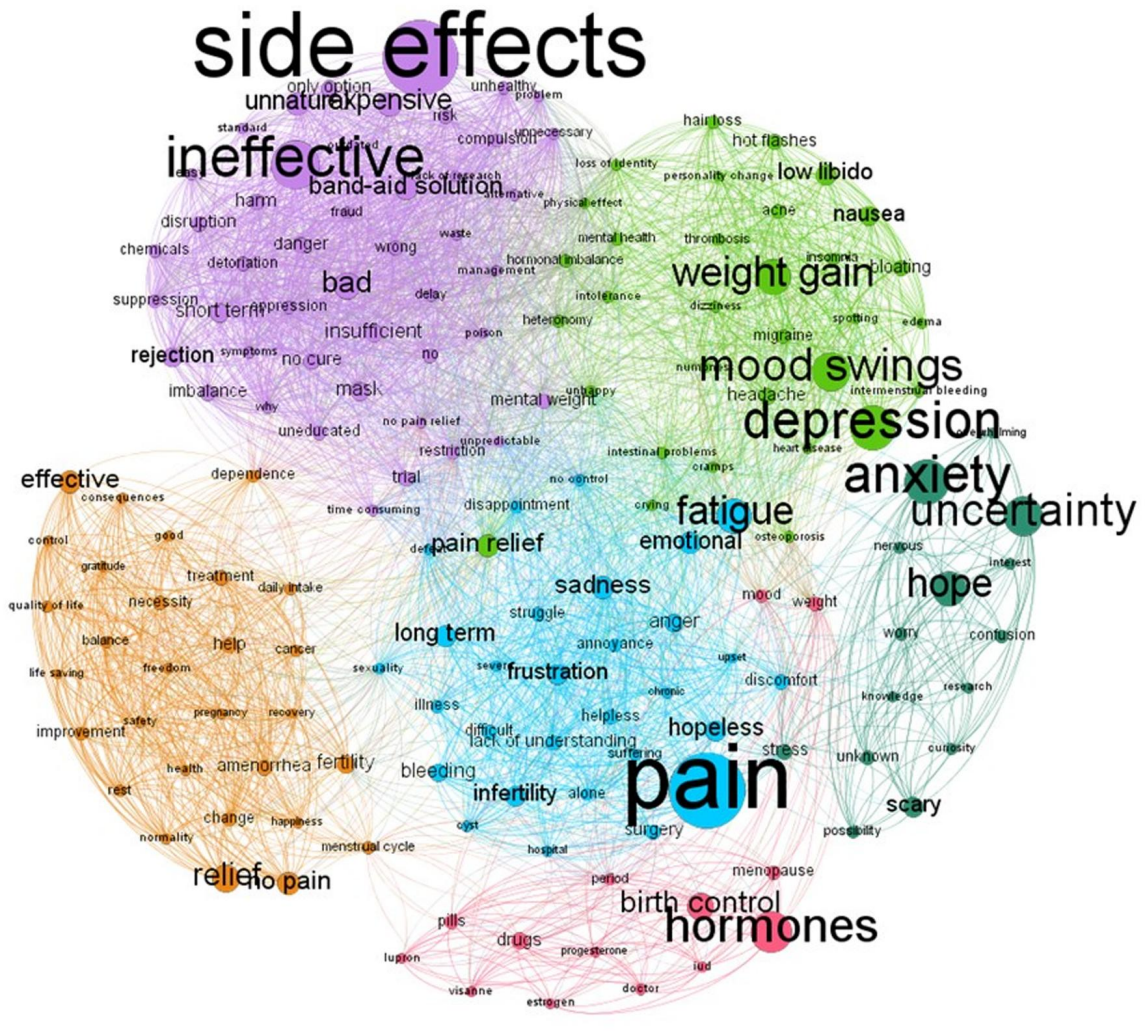
**Free-word association networks to ‘endocrine therapy’ in patients with endometriosis.** Modules emerged from the co-occurrence network of associations. Six modules were identified, and each module was labelled based on its associations: Module 1. List of side effects (grass green); Module 2. Subjective negative factors of rejection (blue); Module 3. Objective negative factors of rejection (purple); Module 4. Positive factors of the treatment (orange); Module 5. Fear from the treatment (dark green); Module 6. Factors related to pregnancy (pink).

**Figure 5.**
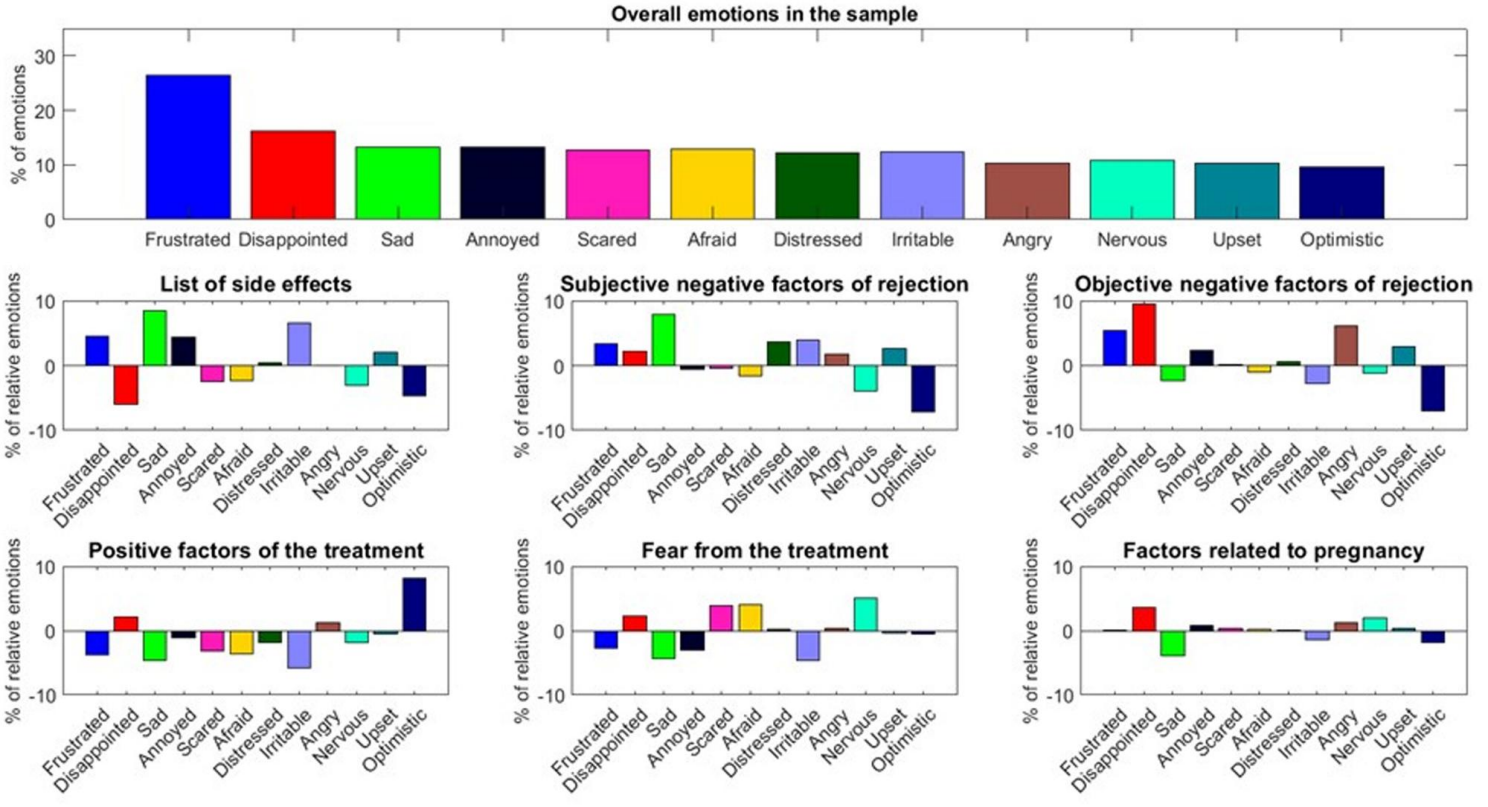
**Distribution of the emotional labels. Emotional labels were derived from the association’s task.** Only the 12 most frequent associations are present. The upper subplot presents the percentage distribution of the emotional labels in the sample. The lower six subplots present the relative percentage distribution of the emotional label in each module. The relative percentual distribution was calculated by eliminating the percentual distribution of the overall sample from each module.

Six separate modules emerged from the co-occurrence of the associations (Fig. 4) with different dominant emotional labels (Fig. 5). Each module was named based on the content of the corresponding associations. Module 1 (mean weight, *M* = 0.17, SD = 0.241) was labelled as the List of side effects, enumerating most of the adverse effects linked to endocrine therapy (most frequent associations: ‘depression’ and ‘mood swings’). Module 2 (*M* = 0.216, SD = 0.251) was labelled as the Subjective negative factors of rejection (most frequent associations: ‘pain’ and ‘fatigue’). Module 3 was the most frequently mentioned (*M* = 0.298, SD = 0.292) and was labelled as the Objective negative factors of rejection (most frequent associations: ‘side effects’, ‘ineffective’). In Module 3, side effects are one critical facet along with other considerations such as treatment inefficacy and costs. Module 4 (*M* = 0.111, SD = 0.197) was labelled as the Positive factors of the treatment (most frequent associations: ‘relief’ and ‘no pain’). Module 5 (*M* = 0.125, SD = 0.21) was labelled as Fear from the treatment (most frequent associations: ‘anxiety’, ‘uncertainty’). Module 6 was the less frequently mentioned (*M* = 0.079, SD = 0.167) and was labelled as the Factors related to pregnancy (most frequent associations: ‘hormones’, ‘birth control’).

Respondents affiliated with Module 1 had a lower level of education (*P* = 0.005, ρ = −0.086). Moreover, they used internet platforms (*P* = 0.008, ρ = 0.082) more often and rated books (*P* = 0.002, ρ = 0.095) as especially useful.

We found no significant associations to Module 2.

Module 3 was associated with a higher level of education (*P* = 1.529e^−07^, ρ = −0.16) and better information about endocrine therapy (*P* = 1.588e^−11^, ρ = 0.204). These respondents found healthcare providers (*P* = 5.415e^−11^, ρ = −0.2) and friends and family (*P* = 0.0002, ρ = −0.111) not useful for providing information. They were less likely to do endocrine therapy (*P* = 0.001, ρ = −0.064) and had less interest in future endocrine therapy (*P* = 3.05e^−15^, ρ = −0.238).

Participants that belonged to Module 4 suffered from less severe symptoms of endometriosis (*P* = 1.614e−05, ρ = −0.132), and the symptoms had a smaller effect on their everyday life (*P* = 0.0005, ρ = −0.107). They were more often doing endocrine therapy at the moment of participation (*P* = 2.2e^−16^, ρ = 0.153) and more interested in future endocrine therapy (*P* = 2.2e^−16^, ρ = 0.267). They used health care providers (*P* = 0.0002, ρ = −0.115) and books (*P* = 0.009, ρ = −0.08) as a source of information less often. Healthcare providers (*P* = 1.445e^−06^, ρ = 0.147) were considered especially useful for providing information about endocrine therapy. Nonetheless, they considered themselves less informed about endocrine therapy (*P* = 0.0006, ρ = −0.105).

Module 5 was significantly associated with lower information about endocrine therapies (*P* = 0.0002, ρ = −0.114). Additionally, these respondents were more interested in future endocrine therapy than other subgroups (*P* = 8.394e^−07^, ρ = 0.15). Respondents affiliated with Module 6 were more likely to do endocrine therapy at the moment (*P* = 7.287e^−05^, ρ = 0.0733) and had significantly more interest in future endocrine therapy (*P* = 0.002, ρ = 0.096).

## Discussion

Endometriosis is a chronic, inflammatory and hormone-dependent disease. Especially hormone dependency, therefore, offers a therapeutic target. In the current international guidelines, endocrine therapies are indicated as the first-choice drug option. They all lead to a clinically significant reduction in pain compared to a placebo (Zondervan *et al.*, 2020; Becker *et al.*, 2022).

This study shows that negative emotions and associations clearly predominate in the population studied. At the same time, there seems to be a lack of information on the part of the medical profession. According to our study, alternative sources, especially social media, are more popular and used more often to obtain information about endometriosis treatment.

Concerning endocrine therapies, among our survey population, only 14.1% (472) of all participants have never done endocrine therapy for endometriosis, and these respondents were mostly not interested at all in doing endocrine therapy in the future. Most affected women, however, come into contact with these therapeutics during their illness.

Our finding that endocrine drugs for endometriosis are associated with negative mental images and emotions replicate the findings of our recent study upon which our questionnaire was based (Burla *et al.*, 2021). Alternative treatment options would be preferred. Some newer therapeutics such as GnRH antagonists are often marketed as ‘new’ and innovative, but they use the same pharmacokinetic mechanism of hypoestrogenism as all endocrine endometriosis drugs (Vercellini *et al.*, 2019). Unfortunately, no promising new therapeutic approach is to be expected in the coming decade (Groothuis and Guo, 2018; Burla *et al.*, 2021). To exit this cul-de-sac, it is essential to understand the reasons for patients’ repulsion of hormonally active drugs. We found that endocrine therapies are very often associated with side effects. Indeed, due to their hormonal activity, these therapies have a wide range of side effect profiles. Nausea, headache, weight gain, skin blemishes, loss of libido, irregular menstrual bleeding, psychological effects, bone loss, thrombosis risk, and impaired fertility are commonly reported adverse effects (Rafique and Decherney, 2017). Among our survey population, psychological side effects such as mood swings or depression were the most popular reason for deciding against endocrine therapy. Apart from ‘side effects’ and ‘depression’, ‘uncertainty’ and ‘ineffective’ were the most frequently named terms in association with endocrine endometriosis therapy. Despite a considerable percentage of non-responders, endocrine endometriosis therapies, more often than not, lead to the amelioration of symptoms (Brown & Farquhar, 2015; Rafique and Decherney, 2017). So there seems to be an information deficit about endocrine therapy. More than a quarter of our study participants stated that they were insufficiently informed about hormonally active drugs (27.09% (907)). Naturally, one would think that healthcare providers would be the main source for endometriosis patients gaining information about treatment options. But our study showed that much information is obtained from alternative sources. As the survey was distributed using social media platforms, it is unsurprising that it was the most frequently used source to gather information about endocrine endometriosis therapies.

Additionally, more and more patients use these platforms to find support, advice, and a sense of community. Many endometriosis-related social media groups and influencers can be found on various platforms; some communities have up to 100 000 members. Fellow endometriosis sufferers are frequently referred to as ‘endometriosis sisters’ or ‘endo sisters’. As this term suggests, exchange, support, and unity are in focus. Interestingly, social media were rated the most useful sources of information by our study population, leaving assumingly more trustworthy sources like health care providers and books behind. Healthcare providers should be aware of social media's massive impact on patients’ views and perceptions of treatment options. They could and should likewise use social media for educational purposes, trust-building, and connecting with patients (Barreto and Whitehair, 2017).

Regarding emotional labels of the associations made by our study population, it stands out that out of the 12 most frequently chosen emotional labels, 11 were negative (Supplementary Fig. S2). This confirms the finding of our recent study about the negative image of endocrine endometriosis therapies (Burla *et al.*, 2021).

It is a fact that the available endocrine drugs are very effective in many cases, which also has been scientifically proven. The different forms of administration, such as pills, implants, patches, injections, etc., should also contribute to the fact that one could build up an individual therapy regime and find the suitable preparation in a suitable form of administration for each patient. Nevertheless, the acceptance rate is low. An earlier study in German-speaking Europe showed that younger, well-educated and urban women, in particular, currently have great reservations about endocrine drugs. Two out of three respondents in that study (66.1%) were dissatisfied with the available hormonal drug options, mostly due to their unfavourable side effects (85.5%), change of libido (67.5%), hormonal cycle disruptions (65.9%), and insufficient alleviation of symptoms (38.2%) (Burla *et al.*, 2021). In the present study, these data have now been confirmed in an international setting. The mostly negative images associated with this could be graphically depicted impressively in our study.

Social media play an increasingly important role in today’s world. It is clear that many of the young, digitally savvy women also get their information from these channels. However, the frequency that our study now reveals is astonishing. The fact that many women do not feel sufficiently informed by their doctors must be of notice. Suppose those affected women are now more likely to obtain information from social media than from specialists. In that case, it cannot be surprising that endocrine preparations are partly judged with negative emotions.

It must be noted that posts on social media tend to be negative and can, therefore, give a distorted picture of reality. It is assumed that Internet and social media users, in particular, seek out information that fits their moods and perception. It is a fact that people are more likely to post when they have something to complain about than when everything is going normally and well.

There is no way to sugarcoat it; the contrast between current guidelines recommending endocrine medications as the primary treatment option and the patients' rejection in this study presents a significant problem. Additionally, the dominance of social media as a primary information source, compared to doctors, is worrisome and can be seen as a failure on our part. Relying solely on social media for information can lead to problems, as negative experiences can spread widely among peers.

Understanding the individual reasons for rejection including potential side effects, and therapy response is crucial. Factors like frustration from dealing with a chronic illness, delayed diagnosis, and worries about the future can play a role.

To address this issue, physicians and medical societies should become familiar with social media discussions about the disease, even considering engagement. Social media hold a prominent place among the younger population, and patients might feel disregarded if doctors are unaware of commonly discussed terms like ‘Endobelly’.

As caregivers for chronic conditions where patients depend on these medications for years, it is our responsibility to provide close guidance and support. In our opinion, proactive education of patients by physicians is therefore mandatory. You must actively point out possible side effects and increase patient visits during the initial period, which is becoming increasingly difficult in an increasingly economically oriented medicine. The efficacy and side effect profiles of these endocrine therapeutic options are highly individual, and unfortunately, finding the right therapy often requires trial and error. This circumstance must be considered, which is why education, in particular, is a key issue. Additionally, it is important to have open discussions with patients to clarify the negative connotations associated with ‘endocrine or hormonal’ therapies. Ultimately, we must reclaim our role as information providers and, if necessary, utilize digital tools for this purpose.

This study has limitations. The translation of the questionnaire might have affected the comprehension of the survey questions depending on the language. We used social media channels to distribute our survey and therefore targeted digitally literate patients. Although endometriosis patients are mostly young, the survey population might not be representative as critical/unhappy patients are more likely to turn to peer groups for advice. Further studies would be important to understand how far our results can be generalized.

## Conclusion

In conclusion, our study shows that endocrine endometriosis therapies are associated with negative mental images and emotions in the subpopulation investigated here. In addition, there seems to be a pre-therapeutic information deficit on the part of physicians. Much information is obtained from alternative sources, such as social media.

Careful education before therapy should be taken seriously, and patients’ concerns should be addressed individually. This could help reduce misunderstandings and misinformation and improve adherence and treatment satisfaction. Given the prevalence of endometriosis and the few emerging pharmaceutical alternatives, these data suggest that further research and development of non-hormonal drugs for treating endometriosis would be favourable.

## Supplementary Material

dead221_Supplementary_Figure_S1

dead221_Supplementary_Figure_S2

dead221_Supplementary_Data_File_S1

## Data Availability

The data underlying this article will be shared on reasonable request to the corresponding author.
