# Supplementary material for: Attitudes and perceptions of affected women towards endocrine endometriosis therapy: an international survey based on free-word association networks
Source: Hum Reprod. 2023 Oct 25;39(1):83–92. doi: 10.1093/humrep/dead221 (PMC10767788; doi:10.1093/humrep/dead221)
Supplement: dead221_Supplementary_Figure_S1 [file dead221_supplementary_figure_s1.pdf]

OXFORD
human reproduction
Human Reproduction, 2023, 1–1
https://doi.org/10.1093/humrep/dead221
Supplementary Data

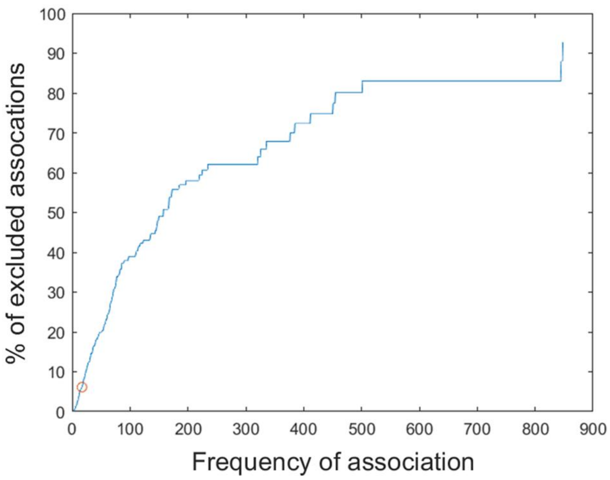

Supplementary Figure S1. Cumulative number of excluded associations. Percentage of excluded associations from the sample as a function of the frequency of individual association. The red circle denotes the first fracture in the function at the frequency with 17 and the percentage of excluded associations 5.4%. That means that excluding associations mentioned only 17 times in the whole sample, only 5.4% of the total produced associations were excluded from the analysis.
