# Supplementary figures and images for: Attitudes and perceptions of affected women towards endocrine endometriosis therapy: an international survey based on free-word association networks
Source: Hum Reprod. 2023 Oct 25;39(1):83–92. doi: 10.1093/humrep/dead221 (PMC10767788; doi:10.1093/humrep/dead221)

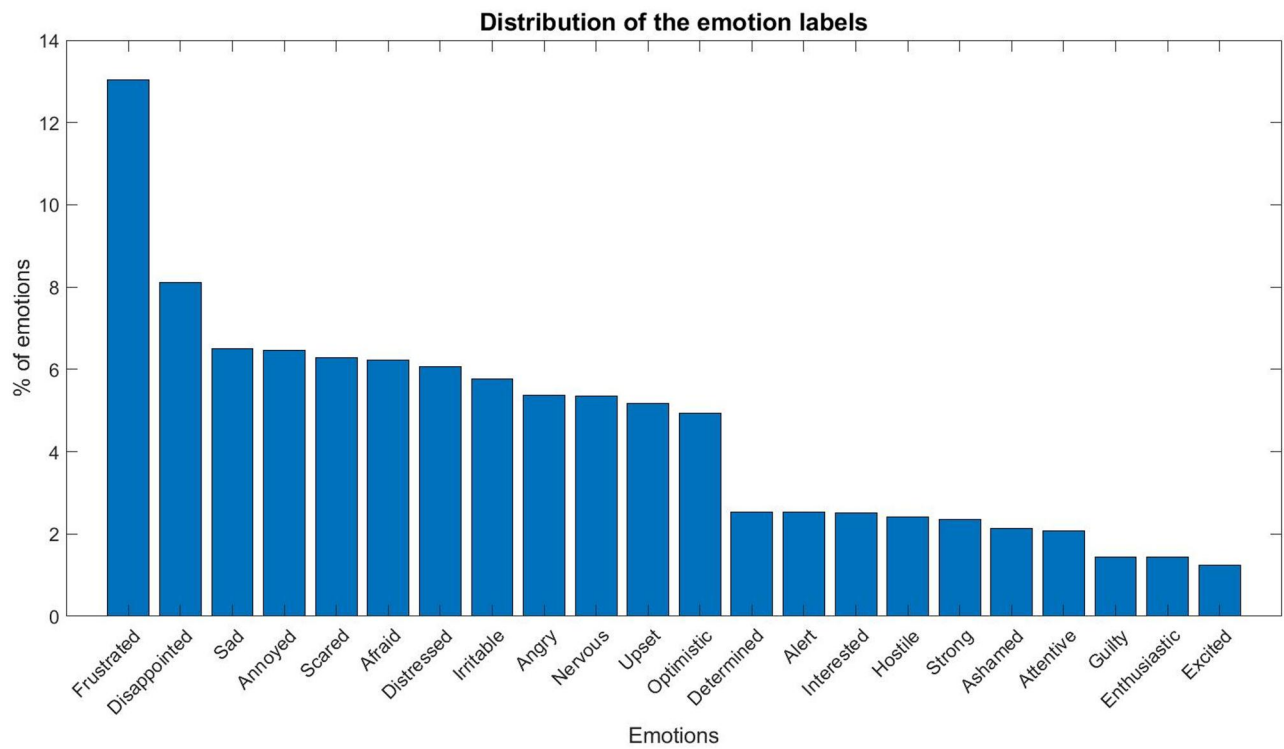

**Supplementary Figure S2.** Frequency distribution of the emotion labels.

Supplement: dead221_Supplementary_Figure_S2 [file dead221_supplementary_figure_s2.pdf]
