# Supplementary material for: Attitudes and perceptions of affected women towards endocrine endometriosis therapy: an international survey based on free-word association networks
Source: Hum Reprod. 2023 Oct 25;39(1):83–92. doi: 10.1093/humrep/dead221 (PMC10767788; doi:10.1093/humrep/dead221)
Supplement: dead221_Supplementary_Data_File_S1 [file dead221_supplementary_data_file_s1.docx]

Supplementary Data File S1: Full questionnaire.

| **Q1.** *How did you get diagnosed with endometriosis?*   1. Histological diagnosis (laparoscopy with biopsy) 2. Suspicion or diagnosis by a gynaecologist (symptoms ± ultrasound) 3. Suspicion or diagnosis by a family doctor (symptoms ± ultrasound) 4. I have never been diagnosed by a doctor (🡪 end of the survey) |
| --- |
| **Q2.** *What are the first 5 words that come to your mind when you think of endocrine treatment for endometriosis?*   1. Word 1 2. Word 2 3. Word 3 4. Word 4 5. Word 5 |
| **Q3-Q7**. *Think about endocrine therapy! Choose TWO states from the list below, which best describe your feelings towards the following expression: Word 1 -Word 5*   1. Attentive, Hostile, Irritable, Alert, Ashamed, Excited, Guilty, Enthusiastic, Distressed, Determined, Upset, Scared, Afraid, Interested, Strong, Nervous, Sad, Frustrated, Angry, Disappointed, Optimistic, Annoyed |
| **Q8.** *How many years have you suffered from symptoms of endometriosis before the diagnosis?* |
| **Q9.** *How many doctors/clinics have you consulted due to your symptoms before obtaining the diagnosis of endometriosis?* |
| **Q10**. *Have you taken hormonally active (endocrine) drugs for endometriosis in the past, or are you taking them currently?*   1. Yes 2. No |
| **Q11.** *Which hormonally active endometriosis medications have you taken so far? (several answers possible):*   1. Combined contraceptives (e.g., ”contraceptive pill”, birth control patch, NuvaRing®) 2. Progestins/gestagens (e.g. Desogestrel (Cerazette®)) 3. Dienogest (Visanne®, Visanette®) 4. GnRH analogues (e.g. Goserelin(Zoladex®)) 5. GnRH antagonists (e.g. Elagolix (Orilissa®)) 6. Aromatase inhibitors (e.g. Anastrozol (Arimidex®), Letrozol (Femara®)) 7. Danazol 8. Long-acting gestagens (Implanon, Depo-Provera®, Intrauterine device containing levonorgestrel (e.g. Mirena®, Jaydess®, Kyleena®)) |
| **Q12.** *How severely do you feel the symptoms of endometriosis on a scale from 1 to 10?* |
| **Q13.** *To what extent do the symptoms of endometriosis usually affect your everyday life on a scale from 1 to 10?* |
| **Q14.** *Are you currently doing endocrine therapy?* |
| **Q15.** *Are you interested in doing endocrine therapy in the future?* |
| **Q16.** *Please rate the importance of these reasons in deciding against endocrine therapy:*   \|  \| Extremely important \| Important \| Moderately important \| Slightly important \| Not important \| \| --- \| --- \| --- \| --- \| --- \| --- \| \| Changes in sexuality (e.g. loss of libido) \|  \|  \|  \|  \|  \| \| Change of menstrual cycle \|  \|  \|  \|  \|  \| \| Psychological effects (e.g. mood swings, depression) \|  \|  \|  \|  \|  \| \| Daily intake \|  \|  \|  \|  \|  \| \| The fact that they are hormonally active \|  \|  \|  \|  \|  \| \| Weight gain \|  \|  \|  \|  \|  \| \| Bloating \|  \|  \|  \|  \|  \| \| Fear of cancer (e.g. breast cancer) \|  \|  \|  \|  \|  \| \| Skin blemishes (e.g. spots, acne) \|  \|  \|  \|  \|  \| \| Thrombosis risk \|  \|  \|  \|  \|  \| \| Osteopenia/osteoporosis \|  \|  \|  \|  \|  \| |
| **Q17.** *Please rate the importance of these reasons in deciding in favour of endocrine therapy:*   \|  \| Extremely important \| Important \| Moderately important \| Slightly important \| Not important \| \| --- \| --- \| --- \| --- \| --- \| --- \| \| Improvement of symptoms \|  \|  \|  \|  \|  \| \| Improvement of quality of life \|  \|  \|  \|  \|  \| \| Lack of alternatives \|  \|  \|  \|  \|  \| |
| **Q18.** *In your opinion, to what extent are you informed about endocrine therapy?*   1. Not at all 2. To a small extent 3. To a moderate extent 4. To a great extent 5. To a very great extent |
| **Q19.** *How often do you use the following sources of information about endocrine therapy?*   \|  \| Daily \| Weekly \| Monthly \| Every 6 months \| Never \| \| --- \| --- \| --- \| --- \| --- \| --- \| \| Healthcare providers (e.g. doctors/nurses) \|  \|  \|  \|  \|  \| \| Social media \|  \|  \|  \|  \|  \| \| Internet/news portals (e.g. Google) \|  \|  \|  \|  \|  \| \| Books \|  \|  \|  \|  \|  \| \| Friends and family \|  \|  \|  \|  \|  \| |
| **Q20.** *How useful do you rate the following sources of information regarding endocrine therapy?*   \|  \| Extremely useful \| Useful \| Moderately useful \| Slightly useful \| Not useful at all \| \| --- \| --- \| --- \| --- \| --- \| --- \| \| Health care providers \|  \|  \|  \|  \|  \| \| Social media \|  \|  \|  \|  \|  \| \| Internet/news portals (e.g. Google) \|  \|  \|  \|  \|  \| \| Books \|  \|  \|  \|  \|  \| \| Friends and family \|  \|  \|  \|  \|  \| |
| **Q21.** *Have you ever been pregnant (including miscarriages and abortions)?*   1. Yes, I have been pregnant (List of Numbers) times. 2. No |
| **Q22.** *Did you ever give birth (vaginal birth or caesarean section)?*   1. Yes, I gave birth (List of numbers) times. 2. No |
| **Q23.** *Age.* |
| **Q24.** *Highest level of education.*   1. Less than a high school 2. Trade School 3. High School 4. Bachelor's Degree 5. Master's Degree 6. Ph.D., other doctorate, or higher 7. Other |
| **Q25.** *Country of residence.* |
| **Q26.** *Do you live in a city or a smaller community/in the countryside?*   1. Smaller municipality, countryside, village 2. City 3. Large city |
| **Q27.** *Religion*   1. Christian (Catholic/protestant or any other Christian denomination) 2. Buddhist 3. Hindu 4. Muslim 5. Jewish 6. No Religion 7. Other religion |
| **Q28.** *Which of the following best represents your ethnicity:*   1. Caucasian 2. Latino/Hispanic 3. Middle Eastern 4. African 5. Caribbean 6. South Asian 7. East Asian 8. Mixed 9. Other |
| **Q29.** *Current relationship status*   1. Single 2. In relationship 3. Married or in a cohabiting relationship 4. Widowed 5. Divorced 6. Separated 7. Other |
| **Q30.** *Do you have any comments?* |
